# 

**Published:** 1917-07-07

**Authors:** 


### Passing Events and Topics.

### Another Mansion as Hospital.

Lord and Lady Rochdale have converted their Keswick seat, Lingholm, a lovely mansion perched on an eminence on the shores of Derwentwater, and surrounded by beautiful grounds, into a V.A.D. hospital. Lady Rochdale is acting as the Commandant.

### The late Lord Masham.

Lord Masham, the gross value of whose estate is £1,016,150, provided in his will that before burial a medical man should perform some surgical operation on his body and certify that life was extinct.

### Removal of Board of Education Offices.

The transfer of the offices of the Board of Education to a part of the premises of the Victoria and Albert Museum will, it is hoped, be complete by Monday, July 9. The public entrance to the offices will be from Exhibition Road.

Rates of Subscription: Twelve months, 6/6; Six months, 4/-; three months, 2/-, post free to any address in the United Kingdom. Abroad, Twelve months, 8/8; Six months, 5/-; Three months, 2/6, post free. **THE HOSPITAL "Index" to Volume LXI., October 1916 to March 1917, is now ready, price 2½d. per copy, post free.** Address The Manager, The Hospital, "The Hospital" Building, 28/29 Southampton Street, Strand, London, W.C. 2.

